# Supplementary material for: Bioactivity-Guided Fractionation and Identification of Antidiabetic Compound of Syzygium polyanthum (Wight.)’s Leaf Extract in Streptozotocin-Induced Diabetic Rat Model
Source: Molecules. 2022 Oct 12;27(20):6814. doi: 10.3390/molecules27206814 (PMC9609764; doi:10.3390/molecules27206814)
Supplement: Supplementary file 1 [file molecules-27-06814-s001.zip › molecules-1869808-supplementary.pdf]

Supplementary Materials

Bioactivity-Guided Fractionation and Identification of Antidiabetic Compound of *Syzygium polyanthum* (Wight.)'s Leaf Extract in Streptozotocin-Induced Diabetic Rat Model

Tri Widyawati [1,2,*], Nor Adlin Yusoff [3,*], Idris Bello [2], Mohd Zaini Asmawi [2] and Mariam Ahmad [2]

1 Pharmacology and Therapeutics Department, Medical Faculty, Universitas Sumatera Utara, Medan, 20155, Indonesia,
2 Department of Pharmacology, School of Pharmaceutical Sciences, Universiti Sains Malaysia, Penang, 11800 Malaysia; idrisbello1@yahoo.com (I.B.); amzaini@usm.my (M.Z.A.); mariam@usm.my (M.A.)
3 Department of Toxicology, Advanced Medical and Dental Institute, Sains@Bertam, Universiti Sains Malaysia, Penang, 13200 Malaysia
* Correspondence: tw_rozan@yahoo.com or tri.widyawati@usu.ac.id (T.W.); noradlinyusoff@usm.my (N.A.Y.); Tel.: +62-8126-582-932 (T.W.); Tel.: +60-111-777-7592 (N.A.Y.)

Methodology

Effects of different doses of squalene in STZ-induced diabetic rats

Thirty-six diabetic rats were divided into six groups (n = 6). Four groups were treated respectively with the following doses of squalene (SQ); 20 mg/kg B.W., 40 mg/kg B.W., 80 mg/kg B.W., and 160 mg/kg B.W. The fifth group received metformin (500 mg/kg B.W.) and served as the positive control. The sixth group was treated with saline (10 mL/kg B.W.) and served as the negative control. All treatments were given orally twice daily for 6 days. Fasting blood glucose levels were measured before (day 0) and 6 days after treatment.

Results

As shown in Figure S1, 20 mg/kg of SQ, administered twice daily for 6 days, had no significant effect on the BGL of diabetic rats when compared with the pre-treatment levels. However, higher doses of SQ, 40, 80, and 160 mg/kg, were shown to reduce BGL significantly ($P < 0.05$–0.001). The observed effect was comparable to metformin (500 mg/kg).

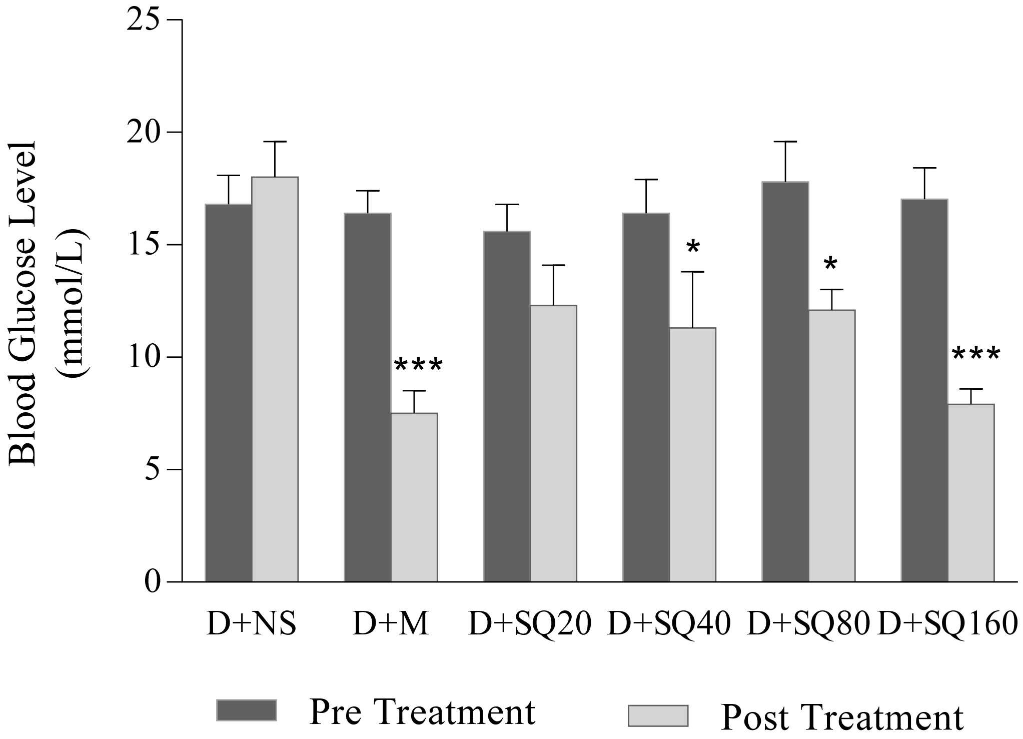

Figure S1. Effects of squalene, administered twice daily for 6 days, on the blood glucose levels of STZ-induced diabetic rats. D + NS, D + M, D + SQ 20, D + SQ 40, D + SQ 80 and D + SQ 160 referred to diabetic rats treated with normal saline (NS), metformin 500 mg/kg B.W. (M), and squalene (SQ) at the doses of 20 mg/kg, 40 mg/kg, 80 mg/kg and 160 mg/kg, respectively. The values are expressed as mean ± SEM (n=6). Pre-treatment and post-treatment differences of BGL were analyzed by using the paired *t*-test, * $P < 0.05$; ** $P < 0.01$;*** $P < 0.001$.
